# Design and Development of a Sprayable Hydrogel Based on Thermo/pH Dual-Responsive Polymer Incorporating *Azadirachta indica* (Neem) Extract for Wound Dressing Applications

**DOI:** 10.3390/polym17152157

**Published:** 2025-08-07

**Authors:** Amlika Rungrod, Arthit Makarasen, Suwicha Patnin, Supanna Techasakul, Runglawan Somsunan

**Affiliations:** 1 Laboratory of Organic Synthesis, Chulabhorn Research Institute, Bangkok 10210, Thailand; amlika@cri.or.th (A.R.); arthit@cri.or.th (A.M.); suwicha@cri.or.th (S.P.); 2 Department of Chemistry, Faculty of Science, Chiang Mai University, Chiang Mai 50200, Thailand; 3 Center of Excellence in Materials Science and Technology, Chiang Mai University, Chiang Mai 50200, Thailand

**Keywords:** sprayable hydrogel, thermo/pH responsive, Pluronic F127, *N*-succinyl chitosan, *Azadirachta indica* (neem)

## Abstract

Developing a rapidly gel-forming, in situ sprayable hydrogel with wound dressing functionality is essential for enhancing the wound healing process. In this study, a novel sprayable hydrogel-based wound dressing was developed by combining thermo- and pH- responsive polymers including Pluronic F127 (PF127) and *N*-succinyl chitosan (NSC). NSC was prepared by modifying chitosan with succinic anhydride, as confirmed by Fourier-transform infrared spectroscopy and nuclear magnetic resonance spectroscopy. The NSC synthesized using a succinic anhydride-to-chitosan molar ratio of 5:1 exhibited the highest degree of substitution, resulting in a water-soluble polymer effective over a broad pH range. The formulation process of the PF127:NSC sprayable hydrogel was optimized and evaluated based on its sol–gel phase transition behavior, clarity, gelation time, liquid and moisture management, stability, and cytotoxicity. These properties can be suitably tailored by adjusting the concentrations of PF127 and NSC. Moreover, the antioxidant capacity of the hydrogels was enhanced by incorporating *Azadirachta indica* (neem) extract, a bioactive compound, into the optimized sprayable hydrogel. Both neem release and antioxidant activity increased in a dose-dependent manner. Overall, the developed sprayable hydrogel exhibited favorable sprayability, appropriate gelation properties, controlled drug release, and antioxidant activity, underscoring its promising translational potential as a wound dressing.

## 1. Introduction

Large surface wounds, such as burns, compromise the skin’s barrier and cause fluid loss, which can lead to organ failure or death. Rapid epithelialization is vital to restore skin function and reduce scarring. Macrophages play a key role in wound healing through their ability to shift phenotypes. In the early phase, pro-inflammatory M1 macrophages clear pathogens by releasing cytokines. As healing progresses, a shift to anti-inflammatory M2 macrophages is essential for tissue repair and promoting angiogenesis and matrix remodeling. However, in chronic or diabetic wounds, this crucial M1-to-M2 transition is often impaired, resulting in prolonged inflammation and delayed healing [1,2].

Hydrogels have extensive applications as ideal artificial extracellular matrix (ECM) materials for supporting in vitro cellular proliferation and differentiation, as well as for in vivo tissue engineering. They have traditionally been used as wound dressings due to their excellent physical characteristics, tunable mechanical properties, and high capacity for drug delivery. Additionally, hydrogels contain a high water content, helping maintain a moist wound environment. In contrast to creams or ointments which only prevent moisture loss, hydrogels actively provide moisture to the wound. This added hydration may reduce infection rates, as moist wounds are associated with a lower risk of infection compared to dry wounds. Usually, severe wounds cause great pain, and pain caused by dressing applications should be taken into consideration. Pain can be exacerbated by manually applying a cream- or ointment-based topical formulation, pressing a dressing into a wound, and subsequently removing a dressing that adheres to the wound [3]. As an alternative, a sprayable hydrogel dressing could alleviate pain associated with wound treatment because it does not require manual spreading or forceful contact with the wound bed. Sprayable hydrogels offer numerous advantages over traditional hydrogels, which are typically used as preformed gel sheets. One key benefit is their ability to be easily sprayed onto irregular surfaces, allowing them closer contact with tissue, thereby forming thin and transparent films that are more conformable to the target area and offer better coverage. They provide a moist environment that supports tissue formation and protects wounds from infection. Furthermore, topical delivery of antibacterial agents makes them great candidates for wound healing [4,5]. Excitingly, ascribed to the ease of use and portability of sprayable hydrogel, it has gradually become an important part of the household medicine cabinet. Recently, the preparation of sprayable hydrogels based on temperature/pH-sensitive polymer has received attention.

In situ gelation by temperature action can be achieved using thermosensitive polymers that form a gel in response to body temperature. Poloxamer 407, also known as Pluronic F127 (PF127) is a thermosensitive polymer capable of forming gels at temperatures close to body temperature. It is a water-soluble copolymer containing polyethylene and polypropylene oxide units. Aqueous solutions of PF127, which are liquid at low temperatures, can self-assemble to form micelles and gradually transform into a semisolid gel form in response to temperature increases. The US FDA has approved the use of PF127 for clinical treatments that are due to its high biocompatibility. To apply the PF127 thermosensitive hydrogel as a spray for skin wound healing, PF127 aqueous solutions, which are low-viscosity liquids, are applied to the wound by a nebulizer and solidify into a semi-solid state once they reach skin temperature. An additional advantage of PF127-based systems is that cooling can revert the gel to a liquid, enabling easy removal during dressing changes and thereby reducing pain. However, PF127 alone has limited bioadhesiveness and moisture-retention capacity. These properties are often improved by blending PF127 with other polymers such as chitosan, Carbopol, or cellulose derivatives [3,6,7].

To introduce pH-responsiveness and improve adhesion, we incorporated chitosan (CS) as a second component alongside PF127. Chitosan is a natural biopolymer known for its biocompatibility and biodegradability, but its limited solubility in neutral aqueous media prompted chemical modification to enhance its water solubility. In this work, *N*-succinyl chitosan (NSC), an acyl derivative of chitosan, was synthesized to serve as a pH-responsive polymer. NSC is water-soluble and highly pH-sensitive due to being rich in reactive functional (-NH_2_, -OH, and -COOH) groups. The protonated amino group (-NH_2_) becomes positively charged (-NH_3_^+^) and interacts electrostatically with negatively charged microbial membrane components, resulting in antimicrobial activity and solubility. Furthermore, the carboxyl (-COOH) and hydroxyl (-OH) groups are reactive sites that can enhance its hydrophilicity, which in effect improves its mucoadhesive properties by forming hydrogen bonds with tissue. Overall, NSC offers the advantages of biocompatibility, biodegradability, and strong bioadhesiveness. The presence of multiple reactive functional groups in NSC also contributes to significant swelling, making it highly suitable for hydrogel-based medical applications [8,9].

Amongst plant sources with a high potential in wound care, *Azadirachta indica*, commonly known as neem, is a widely accepted medicinal plant that is gaining considerable relevance with a large spectrum of biological activity. It is a common home remedy for a number of diseases due to its therapeutic properties, including antibacterial, antifungal, antiviral, anti-inflammatory, antioxidant, anticancer, and immunomodulatory effects. Neem extract has numerous active components such as nimbidin, nimbin, nimbidol, azadirachtin, alkaloids, flavonoids, phenolic compounds, and triterpenoids, promoting the healing of wounds. Neem is also rich in amino acids, vitamins, and minerals essential for tissue regeneration during the wound healing process. According to earlier research, neem exhibits strong antibacterial activity against the common wound pathogens *Staphylococcus aureus* and *Escherichia coli* [10,11]. Consequently, various neem-loaded biomaterials have been explored for wound treatment. For example, electrospun biopolymer nanofibers loaded with neem extract have been developed for use as wound dressings, soft tissue scaffolds, and transdermal carriers [12]. Hydrogels and other matrices incorporating neem have also been investigated, including neem-loaded guar gum/silk fibroin hydrogels for managing chronic wounds [10] and neem-chitosan coated silk sutures for infection control and enhanced wound healing [11]. These studies underscore the promising potential of neem-based formulations in improving wound healing and providing sustained antimicrobial effects.

In light of the above, the aim of this study was to develop a dual pH- and temperature-responsive hydrogel system suitable for a sprayable formulation with potential applications in wound dressing. We designed and optimized a dual-responsive in situ gel formulation using PF127 (thermo-responsive) and NSC (pH-responsive). NSC was synthesized from chitosan and succinic anhydride at varying molar ratios to achieve an optimal degree of substitution and water solubility. The ratio of PF127:NSC was investigated to elucidate the effects of PF127 and NSC concentrations on viscosity behavior and phase transition temperature. Furthermore, the prepared PF127:NSC sprayable hydrogel formulations were evaluated for physicochemical characterization such as gelation time, sprayability, gelling capacity, spreadability, occlusive property, fluid affinity, and stability to determine their suitability as possible wound dressings. To enhance its potential for wound healing applications, neem extract was incorporated into the optimized PF127:NSC sprayable hydrogel formulation. The neem-loaded hydrogel was then evaluated for its in vitro drug release behavior and antioxidant properties. To the best of our knowledge, this study is the first to report a sprayable in situ hydrogel system that simultaneously combines dual responsiveness (to both pH and temperature) with neem-derived bioactive compounds. This unique combination, which has rarely been addressed together in previous studies, provides a multifunctional platform with significant promise for both clinical application and at-home wound management, thereby underscoring the novelty and translational relevance of the present work.

## 2. Materials and Methods

### 2.1. Materials

Chitosan (medium molecular weight), succinic anhydride, Pluronic F127 (PF127), sodium hydroxide, 2,2-diphenyl-1-picrylhydrazyl (DPPH), deuterium oxide (D_2_O), and phosphate-buffered saline tablets were purchased from Sigma-Aldrich, St. Louis, MO, USA. Ethanol, methanol, acetone, hydrochloric acid, and acetic acid were obtained from RCI Labscan. All chemicals and reagents were of analytical grade and were used as received without further purification. Neem extract was obtained from KRUNGTHEPCHEMI Co., Ltd., Bangkok, Thailand. Commercially available spray bottles were bought from the market for use in sprayability experiments.

### 2.2. Synthesis of N-Succinyl Chitosan (NSC)

*N*-succinyl chitosan was prepared according to a reported method with slight modification [8,13]. Briefly, chitosan (0.5 g) was dissolved in 50 mL of 2% *v*/*v* acetic acid and stirred for 1 h at 30 °C. Succinic anhydride (SA) was separately dissolved in 25 mL of methanol at 50 °C and then added dropwise to the chitosan solution under continuous stirring. Three formulations of NSC were synthesized by varying the molar ratio of SA to each glucosamine unit (-NH_2_ group) to 1:1, 3:1, and 5:1, designated as NSC-1, NSC-3, and NSC-5, respectively. After the addition of SA, the reaction mixture was stirred for 24 h at 30 °C. The mixture was then adjusted to pH 7 with 1 M NaOH and stirred at room temperature for 2 h. Then, the product was precipitated with cold ethanol, collected through a centrifuge, dissolved in DI water, and stirred for 18 h. The solution was adjusted to pH 12 with a 1 M NaOH solution. After 24 h, products were first precipitated with cold ethanol, filtered out, and repeatedly washed several times with ethanol and acetone. Finally, the product was dried in an oven for 5 h at 50 °C to obtain the purified NSC as an off-white solid. 

### 2.3. Characterization of NSC

*Structure analysis:* Fourier-transform infrared (FTIR) spectra were recorded using a Fourier-transform infrared spectrometer (Tensor 27, Bruker, Bruker Corporation, Billerica, MA, USA) in the range of 4000–400 cm^−1^ (attenuated total reflectance mode) to identify characteristic functional groups. Moreover, ^1^H-nuclear magnetic resonance (^1^H-NMR) spectra were obtained at 300 MHz using deuterium oxide (D_2_O) as the solvent.

*Solubility:* NSC (20 mg) was dissolved in 15 mL of DI water at room temperature for 24 h under continuous stirring. Then, NSC solutions were adjusted to pH 4, 5, 6, 7, 8, and 9 with 0.1 M HCl and NaOH solutions, and solubility after 6 h was observed.

### 2.4. Preparation of PF127:NSC Sprayable Hydrogel

PF127: NSC sprayable hydrogel formulations were prepared by a cold method to ensure complete dissolution and prevent premature gelation. The numerical part of the sample code represents the %*w*/*v* of PF127 and NSC. For example, 20PF127:0.25NSC represents a hydrogel for which the amounts of PF127 and NSC were 20 and 0.25% *w*/*v*, respectively. To obtain the precursor for the sprayable hydrogel, appropriate volumes of the PF127 solution were mixed with the NSC solution at 4 °C. First, the NSC solution was prepared in water under moderate magnetic stirring for 6 h. Then, PF127 powder was slowly added to the cold NSC solution (4 °C) with gentle magnetic stirring. Thereafter, the polymer solution was left overnight in a refrigerator at 4 °C to ensure complete dissolution of PF127.

### 2.5. Construction of Sol–Gel Phase Transition Diagrams

The sol–gel phase transition was monitored as a function of temperature and pH variation. Gelation was determined by the inverting-tube method, in which a semi-solid state was observed when the vial was flipped and liquid did not flow. The pH values of aqueous hydrogel solutions were adjusted using NaOH and HCl solutions as needed. Hydrogel precursor solutions (approximately 2 mL) were placed in glass vials and kept at room temperature (about 24 ± 1 °C) for 1 h before being heated in a water bath. The temperature was increased in 1 °C intervals and equilibrated for 10 min before dipping the vial in the water bath. When it was observed that the solutions in the vial could no longer flow within 5 s, the corresponding temperature and time were recorded. Optimum ratios of sprayable hydrogels were determined and selected with sol–gel transition temperature of 35 ± 1 °C or below (just below skin temperature) to ensure rapid gelation upon application to the body.

### 2.6. Evaluation of Gel Formulation

#### 2.6.1. Clarity of Formulations

The prepared spray solution was visually inspected for physical appearance and uniformity against a black and white background in terms of turbidity, color, clarity, and the presence of particles. Three grades were assigned for the visual assessment: very clear (glassy) (+++), clear (++), and turbid (+).

#### 2.6.2. Sprayability

The sprayability of the prepared hydrogel solutions was evaluated using conventional spray bottles, each filled with 5 mL of sample. Spray performance was assessed based on several criteria: ease of spraying (including the absence of clogging and smooth trigger operation), uniformity of the spray pattern, and the ability to form a continuous film on a surface. To quantify the amount sprayed, each sample was sprayed 15 times into a beaker, and the total weight of the dispensed solution was measured [5]. In addition, the effect of spray distance on hydrogel distribution was examined by spraying the formulations from distances of 5 cm and 10 cm onto a smooth, horizontally orientated surface. The hydrogel solution, mixed with orange food dye for visibility, was dispensed using manually operated spray bottles. Each spray was applied with the lowest and most consistent manual pressure to ensure a uniform volume per spray. The sprayed patterns were captured on paper, and the spray distribution was analyzed at the specified distances. The spraying efficacy was quantitatively assessed by generating 3D surface maps of the sprayed areas using ImageJ software version 1.54. To minimize errors, the sprayability of each sample was tested in triplicate using three different spray bottles.

#### 2.6.3. Gelling Capacity

The investigations were carried out in accordance with previous studies, with slight modifications [14,15]. The gelling capacity of the hydrogel was assessed by adding one drop of the formulation into phosphate-buffered saline (PBS, pH 7.4) at 34 ± 1 °C. Gelation was confirmed visually by the droplet’s ability to retain its shape without dispersing. The gel strength was qualitatively classified based on the duration of gel stability as follows: (+) gelled after a few minutes but dissolved rapidly; (++) immediate gelation that remained for a few minutes; (+++) immediate gelation that remained for nearly an hour.

#### 2.6.4. Spreadability Test

The hydrogel solution taken from the refrigerator was kept at room temperature for at least 1 h. A quantity of 1 g of hydrogel solution was weighed and placed at the center of a Petri dish, which had previously been marked with a circle of 2 cm in diameter. The dish was then placed onto a hot plate (35 ± 1 °C). Another petri dish was placed over the first, and 500 g of weight was allowed to rest on top. After 1 min, the diameter of the spread area (cm) was measured with a ruler [14,16].

#### 2.6.5. Occlusive Property

The hydrogel solution was sprayed on Whatman filter paper (No. 1) covering a glass beaker containing 50 mL of water. Another beaker filled with 50 mL of water and wrapped in filter paper without a sample represents the control. Both beakers were stored at room temperature for 48 h with 48% relative humidity. The evaporation of water through the membrane was measured based on the reduction in water weight in each beaker. The F value, i.e., the occlusion factor, was calculated using the following equation.

$$F=\frac{\left(A-B\right)}{A}\times 100$$

where *A* is the reduction in water weight from the control beaker and *B* is the reduction in water weight from the sample beaker.

#### 2.6.6. Fluid Affinity

Fluid absorption and donation of hydrogel were tested according to the EU industry standard EN 13726-1:2002 [17], as previously reported [18]. Gelatin (35% *w*/*w*) and agar (2% *w*/*w*) were used in this test to emulate dry and exuding wounds, respectively. Both were prepared in solution A (142 mmol Na^+^, 2.5 mmol Ca^2+^), a wound exudate simulation that emulates the ion concentration of human serum or blood. The gelatin and agar solutions were heated to 60 ± 1 °C for 14 h and 120 ± 1 °C for 1 h, respectively. 10.0 ± 1 g either gelatin solution or the same amount of agar solution was filled into 50 mL syringes with the tip removed. All syringes containing gelatin and agar were covered with Parafilm^®^ to prevent condensation and left to settle for 3 h. Thereafter, 10.0 ± 1 g of hydrogel formulation was added to the syringe after removing the Parafilm^®^. After filling with hydrogel formulation, the syringe was again covered with Parafilm^®^ and kept at 25 ± 2 °C for 48 h. The fluid absorption or donation (% *w*/*w*) of the formulation (*W*_5_) was calculated using the following equation.

$$W_{5}=\left(\frac{\left(W_{3}-W_{4}\right)-\left(W_{2}-W_{1}\right)}{\left(W_{2}-W_{1}\right)}\right)\times 100$$

where *W*_1_ is the weight of the gelatin or agar, *W*_2_ is the weight of the gelatin or agar + test formulation, *W*_3_ is the weight of the gelatin or agar + test formulation 48 h after testing, and *W*_4_ is the weight of formulation-removed agar or gelatin 48 h after testing.

### 2.7. Stability of Solution

The physicochemical stability of the hydrogel precursor solutions was monitored by storing samples at 4 ± 1 °C in the refrigerator for an extended period (up to 4 months) and observing any changes. Key parameters checked included clarity, pH, gelation time, spreadability, and gelling capacity.

pH determination: The pH of the hydrogel solutions was measured using a calibrated digital pH meter (Mettler-Toledo, Greifensee, Switzerland). Samples (5 mL) of each formulation were equilibrated at room temperature, and the pH was recorded. pH readings were taken immediately after preparation and periodically during storage to monitor stability.

### 2.8. Cytotoxicity Assay

A human fetal lung fibroblast cell line (MRC-5) was used to evaluate the cytotoxicity of the 20PF127:0.25NSC sprayable hydrogel. The MRC-5 cells were seeded in 96-well plates at a density of 5000–20,000 cells per well in culture medium (DMEM) and allowed to grow at 37 °C with 95% humidity and 5% CO_2_ for 24 h. The cytotoxicity assay was initiated by adding an equal volume of cell culture medium containing each test polymer at predetermined concentrations. After 48 h, each well received 100 μL of the MTT reagent (0.5 mg/mL, in serum-free cell culture medium) and was further incubated for 2.5–4.0 h at 37 °C with 95% humidity and 5% CO_2_. Subsequently, the medium was replaced with 100 μL of DMSO to dissolve the purple formazan crystals. Thereafter, Spectra-Max Plus 384 microplate readers (Molecular Devices, Sunnyvale, CA, USA) were used to measure the absorbance at 550 nm.

### 2.9. In Vitro Drug Release Studies

Neem-containing formulations: in the first step, PF127:NSC sprayable hydrogel solution was prepared, and the pH was adjusted to 5 using 0.1 M of NaOH and HCl. Then, varying amounts of neem extract (0.05, 0.10, 0.20, and 0.30 g/mL) were added to the polymer solution and stirred at room temperature for 1 h until a homogenous solution was achieved. A total of 2 mL of the formulation was placed into a vial and transferred to a water bath set at 37 °C for gel formation. After gelation occurred, 3.0 mL of phosphate-buffer saline (PBS, pH 7.4), used as the release medium, was added to the vial, which was placed in a water bath set at 37 °C. At predetermined time intervals, 1 mL of the release medium was removed for the measurement, and the same volume of fresh PBS medium was added to replace it. The release medium was filtered using a 0.22 μm membrane filter before the neem extract was quantified using a UV-vis spectrometer (microplate reader, BioTek, Winooski, VT, USA) set at 355 nm [10].

### 2.10. Antioxidant Activity

The antioxidant activity of free neem extract was evaluated based on its scavenging ability against free 2,2-diphenyl-1-picrylhydrazyl (DPPH) radicals. Neem concentrations were prepared in methanol by serial dilution, ranging from 50 to 2000 μg/mL. After that, 100 µL of 0.004% DPPH solution in methanol was added to 0.3 mL of each neem concentration and left in a dark place for 30 min. The change in absorbance of DPPH at 517 nm was measured using a microplate reader [12]. The antioxidant activity of neem extract was calculated using the following equation:

$$\mathrm{Antioxidant}\;\mathrm{activity}\;\left(\%\right)=\frac{\left(A_{C\;}-A_{S\;}\right)}{A_{C\;}}\times 100$$

where *A_C_* is the absorbance of the control, and *A_S_* is the absorbance of the sample.

For the neem-loaded PF127:NSC sprayable hydrogel, a vial containing 2 mL of hydrogel solution was placed in a water bath at 37 °C for gel formation. Subsequently, 3 mL of methanol was added and kept for 2 h, followed by the procedure described above.

### 2.11. Statistical Analysis

To determine the statistical difference, a one-way ANOVA technique followed by a Tukey’s post hoc test was performed using GraphPad Prism 6.0 software. The experiments were repeated three times. The results were expressed as means ± standard deviation (SD), and *p* < 0.05 was considered statistically significant.

## 3. Results and Discussion

### 3.1. Preparation and Characterization of N-Succinyl Chitosan

In this study, chitosan macromolecules were chemically modified using succinic anhydride to obtain *N*-succinyl chitosan (NSC), a water-soluble derivative of chitosan. The modification was carried out in an acid–alcohol medium, where succinic anhydride selectively reacted with the amino groups on the chitosan backbone. This reaction introduced polar carboxyl groups into the chitosan backbone, thereby enhancing its solubility in water.

**Figure 1:**
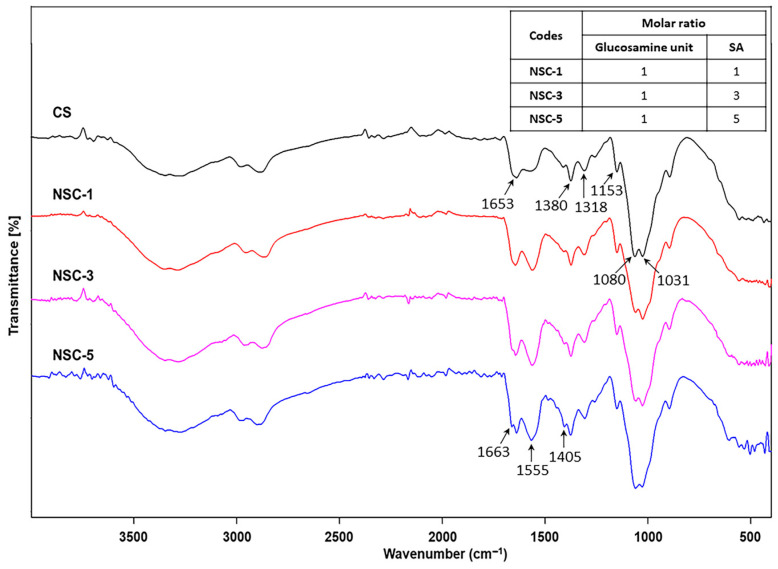
FTIR spectra of chitosan and *N*-succinyl chitosan products (CS = chitosan and SA = succinic anhydride).

*Structures analysis:* The chemical structure of chitosan (CS) and *N*-succinyl chitosan (NSC) were identified by FTIR. Figure 1 presents the FTIR spectra of both CS and NSC, highlighting the structural changes resulting from succinylation. For native chitosan, characteristic absorption peaks were observed as follows: broad O-H stretching between 3100–3600 cm^−1^ and -NH_2_ stretching at 3427 cm^−1^, C-H stretching at 2880 cm^−1^, and the bending vibration of N-H in primary amide at 1653 cm^−1^. Additional peaks included CH_2_ bending symmetrical deformation at 1490 cm^−1^, CH_3_ symmetric angular deformation at 1380 cm^−1^, and the amide III band at 1318 cm^−1^. The characteristic peaks at 1153 cm^−1^ correspond to asymmetric stretching of the C-O-C bridge, and skeletal vibration involving the C-O stretching at 1080 and 1031 cm^−1^ is characteristic of a sugar structure. Upon succinylation, the FTIR spectrum of NSC displayed several notable changes. The broad peak for O-H and N-H stretching shifted to a lower wavenumber and became sharper, appearing at 3252 cm^−1^, indicating changes in hydrogen bonding. A new peak appeared at 1405 cm^−1^, corresponding to the asymmetric stretching of the carboxylate (COO^−^) group, confirming the successful introduction of succinyl moieties. In addition, the original N-H bending band of the primary amide at 1653 cm^−1^ disappeared, and two new bands were observed at 1663 and 1555 cm^−1^ corresponding to C=O stretching and N-H (secondary amide) bending of the succinic group. These results suggest that the amino groups of chitosan were substituted, and that -NH-CO- groups were formed for NSC [8,13,19].

**Figure 2:**
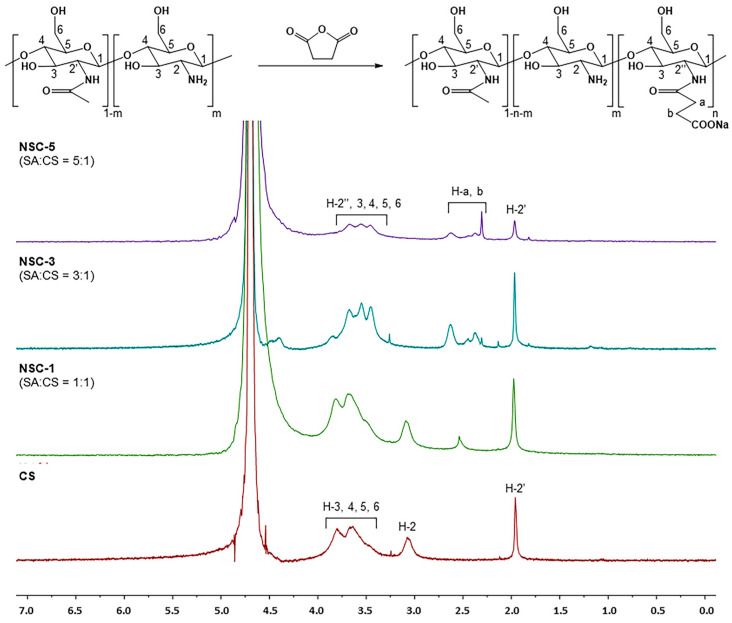
^1^H-NMR spectra of chitosan and *N*-succinyl chitosan products (CS = chitosan and SA = succinic anhydride).

The chemical modification of chitosan was further confirmed by the ^1^H-NMR spectrum of NSC, as shown in Figure 2. Apparently, the native chitosan showed signals at the chemical shifts of 1.96 ppm (methyl protons of the acetyl group, -NH[CO]CH_3_), 3.08 ppm (H-2 of D-glucosamine unit, GlcN), and in the range of 3.68–3.80 ppm (H-3, H-4, H-5, and H-6 of GlcN). Following succinylation, all NSC products exhibited a new set of signals in the range of 2.28–2.63 ppm, which correspond to the methylene protons (-CH_2_-CH_2_-) of the succinyl group (-NHCOCH_2_CH_2_COONa). These additional peaks were present alongside the original chitosan signals, indicating successful grafting of succinyl groups onto the chitosan backbone. The ^1^H-NMR spectral characteristics of the synthesized NSC were consistent with those reported in previous studies [20], confirming the structural identity of the NSC product. The degree of substitution (DS) of the succinyl groups on chitosan was calculated from the NMR integrals using the following equation [21].

$$\mathrm{DS}=\frac{\left(A/4\right)}{\left(A^{\prime }/6\right)}\times 100$$

where *A* represents the integral value of protons corresponding to -CH_2_-CH_2_- (H-a and H-b) of the substituted succinyl group and *A*′ represents the integral value of protons corresponding to H-2″, H-3, H-4, H-5 and H-6 (CS backbones).

As expected, increasing the molar ratio of succinic anhydride (SA) to glucosamine units (or per NH_2_ group) resulted in a higher degree of substitution (DS). The highest DS value was observed for NSC-5 (57.2%), corresponding to a 5:1 molar ratio of succinic anhydride to glucosamine units (or per NH_2_ group). This was followed by NSC-3 (37.3%) at a 3:1 ratio and NSC-1 (11.3%) at a 1:1 ratio; the results are summarized in Table 1.

*Solubility test:* The solubility of CS and NSC products, determined by turbidity measurements of solutions at various pH levels, is presented in Table 1. It was found that NSC-1 cannot dissolve in the pH 5–9 region in a similar manner to neat CS. This was anticipated since the CS chain contained amino groups that were protonated at low pH levels. NSC-3 became partially soluble over the higher pH range due to the increasing substitution of the amino groups by carboxylic groups, which became negatively charged above pH 6. This is also not completely soluble at high pH due to the predominance of amino groups compared to carboxylic groups, resulting in total solubilization at low pH. On the other hand, the high SA content (NSC-5), which is high in DS, can dissolve in high acidic and high basic conditions (i.e., pH ≤ ~5 and pH ≥ ~8, respectively). This phenomenon is in accordance with previous research [13] due to the presence of both primary and/or secondary amino and carboxylic groups on the highly substituted NSC products. Consequently, NSC-5 was used as a component in the sprayable hydrogel preparation combined with PF127.

### 3.2. Construction of Sol–Gel Phase Transition Diagrams

**Figure 3:**
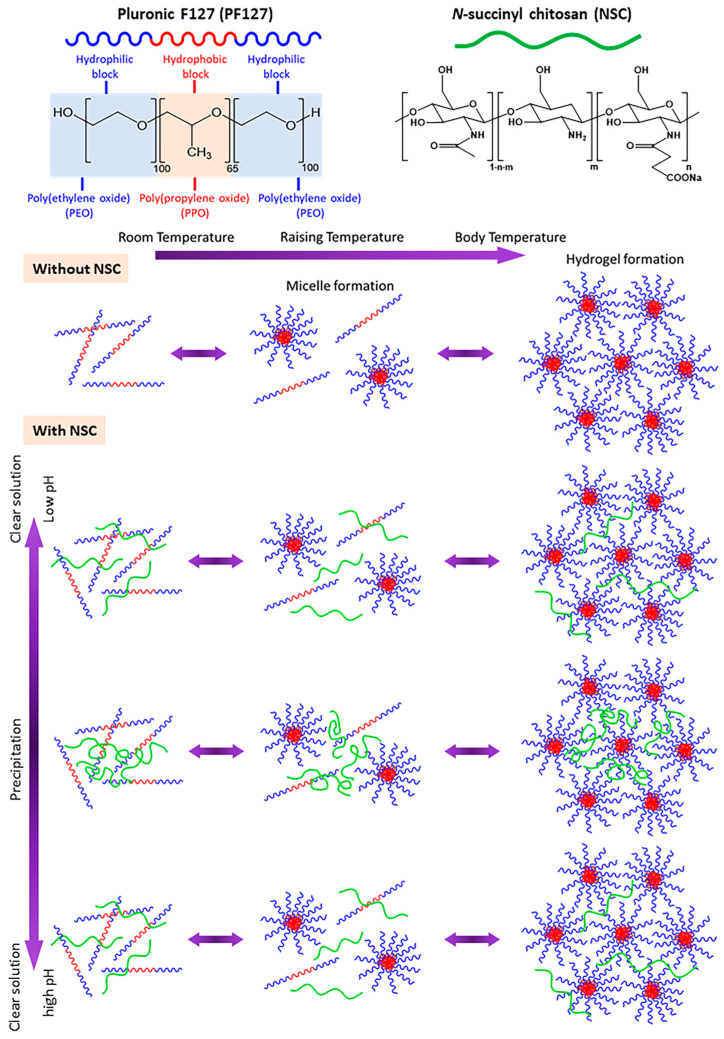
Schematic of in situ gelation mechanism of pH- and thermo-responsive PF127:NSC aqueous solution.

Sprayable dressing formulations must meet two essential criteria: (i) they should be free-flowing liquids at room temperature that can be sprayed into wounded tissue; (ii) they should readily form in situ gels at skin temperature. Therefore, the gelation temperature (T_gel_) is acknowledged as one of the most important parameters for in situ gel-forming systems when these gels are utilized for wound treatment applications [14]. To meet these requirements, a test tube inversion method was used to examine the sol–gel phase behavior of our developed sprayable formulation and determine its T_gel_. In this method, an aqueous polymer solution in a test tube is inverted to observe its ability to flow as a function of temperature. The temperature at which the liquid ceases to flow upon tube inversion, indicating gelation, is referred to as T_gel_. In addition to being regarded as easy, the technique has been used previously to investigate the temperature-dependent phase behavior of a variety of polymers. For this reason, it led to the objective of this study: the development and evaluation of a dual temperature- and pH-responsive compound specifically designed to accommodate the needs of sprayable wound dressings. *N*-succinyl chitosan (NSC) was employed as the pH-responsive polymer, while Pluronic F127 (PF127) served as the thermo-responsive polymer. The formation mechanism of the PF127:NSC sprayable hydrogel at different temperatures and pH levels is illustrated in Figure 3. The gelation mechanism of PF127 involves self-assembly into micelles in aqueous solution. At low temperatures, PF127 molecules are surrounded by a hydrated layer. As the temperature increases, hydrogen bonds between water and the hydrophilic poly(ethylene oxide) (PEO) blocks are disrupted, promoting hydrophobic interactions among the poly(propylene oxide) (PPO) blocks. This results in micelle formation, with PPO cores and PEO shells. As the ambient temperature rises, the growing number and close packing of PF127 micelles result in the gelation process [22]. Additionally, pH variations significantly influence the phase transition behavior of NSC. At low pH, the amino groups of the D-glucosamine units in NSC become ionized. The repulsion between the cationically-ionized backbones expands the polymer chains, resulting in pairwise interactions, or hydrogen bonds, between the molecules of water and NSC polymer. As a result, the hydrogel appears as a clear solution. At neutral pH, deprotonation of amino groups occurs, enhancing intermolecular interactions between NSC chains and reducing water–polymer interactions, leading to precipitation. At higher pH, ionized carboxyl groups increase repulsion between polymer chains, restoring the solubility and clarity of the solution [23].

**Figure 4:**
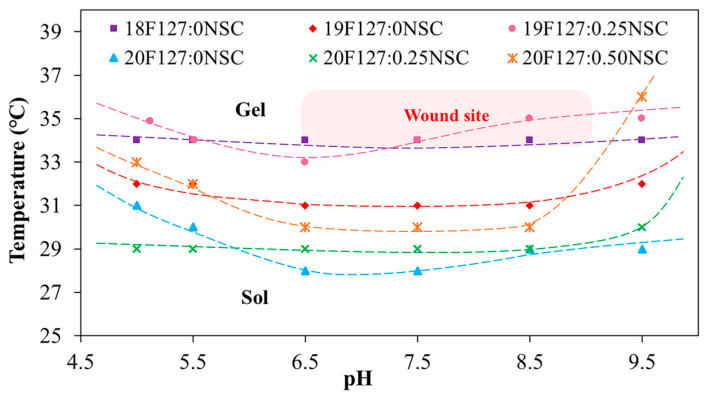
Sol–gel phase transition diagrams plotted between temperature as a function of pH (*x*PF127:*y*NSC indicates a mixture with *x*% *w*/*v* PF127 and *y*% *w*/*v* NSC).

The effects of varying concentrations of PF127 and NSC on the sol–gel phase transition behavior were systematically investigated. The phase diagrams plotting T_gel_ as a function of pH of various PF127:NSC formulations are shown in Figure 4. It was found that sprayable formulations, which are liquid at low temperatures, can transform into a semi-solid gel form in response to temperature increases due to the self-assembly of PF127 into micelles. Based on the previous works [24], a hypothetical scenario can be proposed to illustrate hydrogen bonding between PF127 ether and water molecules and van der Waals interactions present at various temperatures in PF127. At low temperatures (T < LCST), hydrogen bonding between PF127′s ether groups and water predominates, forming a hydration shell that keeps the polymer soluble. As the temperature rises, this balance changes—hydrophobic interactions between polymer chains become more favorable. This induces the transition of PF127 from hydrated loose coils to hydrophobic collapsed chains, altering the structure of the water and releasing water molecules.

At lower concentration of PF127 that can undergo thermo-reversible gelation below 35 °C, which is nearly skin temperature, preliminary investigations were carried out using concentrations ranging from 17 to 20% *w*/*v*. At the lowest PF127 concentration (17% *w*/*v*), no gel formation was observed near skin temperature or within the pH range of 5.0–9.5, likely due to weak intermicellar interactions. Within the same temperature and pH range, PF127 at concentrations of 18–20% *w*/*v* successfully formed gels. Based on these results, a PF127 concentration of 18–20% *w*/*v* was selected as a studied range concentration, which exhibits suitable thermo-reversible properties and is also widely used in previous studies for biomedical materials applications [25]. When the PF127 concentration increases to 19% and 20% *w*/*v* (19F127:0NSC and 20F127:0NSC), the T_gel_ decreases, indicating enhanced micelle formation and stronger intermolecular interactions that facilitate gelation at lower temperatures.

However, the strong impact of salt ions on the LCST transition of the temperature-responsive polymers is a very important subject. Figure 4 demonstrates the impact of the solutions with different pH on the T_gel_ of PF127. Considering pure PF127, in both acid and base solutions, the T_gel_ point rose. It is postulated that in acid solutions, some water molecules in the hydration layer around PF127 become protonated (forming hydronium ions), which disrupts hydrogen bonding between PF127’s ether oxygens and water. At the same time, the cage-like water structures around the hydrophobic parts of PF127 become less stable. For neutral or base solutions, another mechanism can be postulated, with the hydrated layer transformed not only due to interactions between water and the salt anions but also due to the strong association of ethylene glycol groups of the PF127 with the salt cations (Na^+^). Therefore, various buffer types can alter the hydration layer surrounding the polymer, thereby affecting the gelation temperature. A similar effect was previously reported [26] and shows a hypothetical scheme of the behavior of temperature-responsive polymers in the various pH buffer solutions. In previous reported works, the salt ions had a strong influence on the LCST transition of poly(di(ethylene glycol)methyl ether methacrylate), demonstrating the role of ionic species in disrupting hydration layers and altering polymer–water interactions.

**Figure 5:**
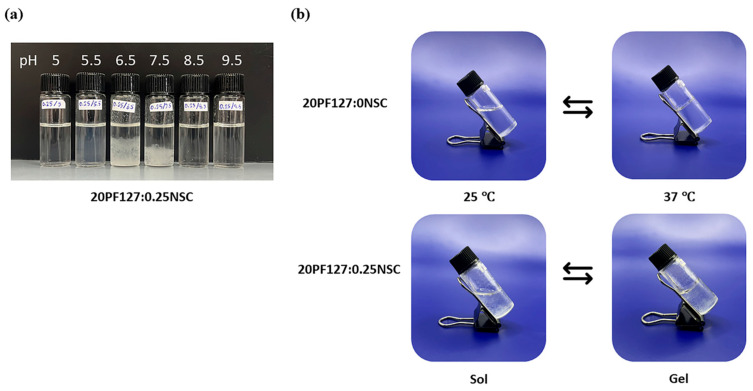
(**a**) Appearances of sprayable hydrogel solution (20PF127:0.25NSC) under different pH; and (**b**) representative photographs of sol and gel states at 25 and 37 °C.

The concentration of NSC at 0.25–0.50% *w*/*v* was combined with PF127 to evaluate its effect on gelation and pH responsiveness. The sprayable formulations with NSC were capable of forming a gel within a pH range of 5.0 to 9.5. However, the physical appearance of the solution varied, showing different levels of clarity at different pH values. Formulations with NSC exhibited precipitation at a pH range of 6.5–7.5, as shown in Figure 5a. Notably, despite visible precipitation, all NSC-containing formulations were still able to form gels within the physiological pH range (6.5–7.5). Figure 5b shows the gelation at pH 7.5 of the representative solution with and without NSC. It was found that the 20PF127:0NSC solution was triggered to form a gel, a translucent semi-solid, when the temperature was raised. 20PF127:0.25NSC solution, which initially appeared as a precipitate, occurs as a gel form that becomes turbid as the temperature increases. These findings suggest that NSC does not inhibit gel formation at high temperature, although it may influence the clarity and physical characteristics of the gel. For instance, formulations containing 0.25% *w*/*v* NSC (19F127:0.25NSC and 20F127:0.25NSC) show lower transition temperatures than those without NSC, particularly at intermediate pH values (6.5–7.5). This trend suggests that NSC promotes micelle packing and network formation, facilitating gelation at lower temperatures.

**Figure 6:**
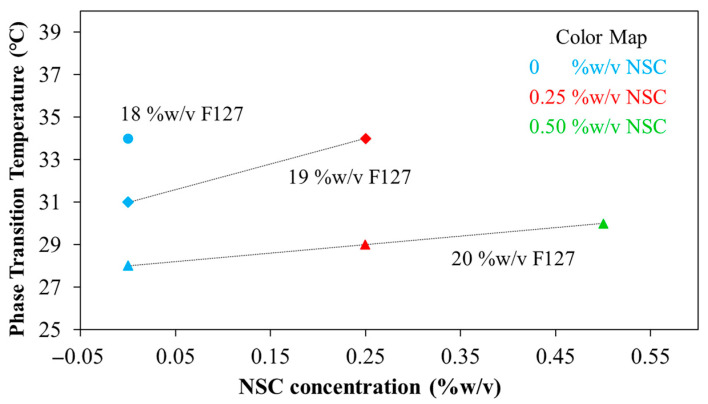
Phase transition temperature at pH 7.4 of the PF127:NSC spray solution with different PF127 and NSC concentrations.

Since the pH of wounded skin typically ranges from 6.5 to 8.9, we evaluated gelation temperatures at pH 7.4. The T_gel_ of the formulations ranged from 28 to 34 °C, depending on PF127 and NSC concentrations (Figure 6). In the formulation without NSC, increasing the PF127 concentration from 18 to 20% *w*/*v* leads to a decrease in T_gel_ from 34 to 28 °C, reflecting the formation of more densely packed gel networks. However, NSC leads to a remarkable rise in the T_gel_. This indicates that the hydrogen bonds in densely-packed PF127 gels are enfeebled by hindering of NSC molecules, thereby increasing the T_gel_ [25]. Specifically, hydrogen bonds are likely formed between the ether oxygen atoms in the PEO chains of PF127 and the hydroxyl and carboxyl groups of NSC. In the sol state, the NSC chains are randomly dispersed due to hydrophobic interactions with the PPO of PF127. Upon increasing the temperature, PF127 micelles self-assemble into a gel network. However, in the presence of a polysaccharide such as NSC, the micelles may become surrounded by polymer chains, potentially disrupting or delaying temperature-induced gelation. This interference likely occurs simultaneously with the hindrance of hydrophobic interactions among intermolecular PF127 molecules by the interspersed NSC chains [27,28]. For formulations containing 18% PF127, the addition of NSC did not cause gelation under the tested conditions, further supporting the hindering effect of NSC on gelation. At 19% PF127, gelation occurred only at low NSC concentrations in the range of 0 to 0.25%, where T_gel_ increased from 31 to 34 °C, while higher concentrations had no gel formation. In contrast, at 20% PF127 successfully formed gels even with NSC concentrations as high as 0.50%. When increasing NSC concentration from 0 to 0.50% *w*/*v*, T_gel_ was increased from 28 to 30 °C. Therefore, an optimal balance between PF127 and NSC is crucial for achieving the desired gelation behavior at physiologically relevant temperatures and pH. The five formulations, i.e., 19PF127:0NSC, 19PF127:0.25NSC, 20PF127:0NSC, 20PF127:0.25NSC, and 20PF127:0.50NSC, successfully gelled across a broad pH range and skin temperature, as confirmed by sol–gel phase transition analysis. According to these findings, the five formulations mentioned above were selected for further evaluation of gel properties to determine their suitability for use as sprayable wound dressings.

### 3.3. Evaluation of Gel Properties

The sprayable hydrogel formulations were evaluated for optimized dressing formulation based on several key characteristics, including clarity, gelation time, sprayability, gelling capacity, spreadability, occlusive properties, and fluid affinity.

#### 3.3.1. Clarity of Formulations

To avoid skin irritation or allergic reactions, topical formulations should maintain a pH compatible with the skin’s natural pH, which typically ranges between 4.0 and 6.0 [29]. The sprayable hydrogel formulations in this study were adjusted to pH 5 to ensure compatibility with skin application. Consequently, the PF127:NSC spray solutions were adjusted to pH 5 using dilute HCl or NaOH, after which other properties were evaluated. All developed sprayable hydrogel formulations were assessed for clarity, demonstrating that the clarity of all solutions was sufficient and solutions were free-flowing (Table 2). However, formulations containing a higher concentration of NSC (0.50% *w*/*v*) exhibited slight turbidity. Overall, the appearance and clarity of all formulations were deemed acceptable for topical application.

#### 3.3.2. Gelation Time

For in situ hydrogel applications, rapid gelation at body temperature is essential to prevent formulation loss after application. If the formulation remains in liquid form, it may easily drain away from the wound site before gelation occurs. The gelation times of the various formulations at 37 °C are summarized in Table 2. It was found that the gelation time ranged from 44 to 362 s, depending on the ratios of PF127 and NSC. Specifically, higher NSC content resulted in prolonged gelation times, while increasing PF127 concentration shortened the gelation time. Increasing the concentration of PF127 reduces the gelation time due to the higher number of triblock copolymer chains, which promote micelle formation and packing. In contrast, the addition of NSC increases the gelation time, as the interspersed NSC chains interfere with hydrophobic interactions between PF127 molecules, delaying micelle aggregation and gel formation. These results indicate that a shorter gelation time is preferable for sprayable wound dressings, as faster gelation helps ensure proper retention and adhesion at the application site. Among all tested formulations, 19PF127:0.25NSC showed the longest gelation time, taking approximately 362 s to form a gel. Such delayed gelation is unsuitable for use as a sprayable wound dressing.

#### 3.3.3. Sprayability

**Figure 7:**
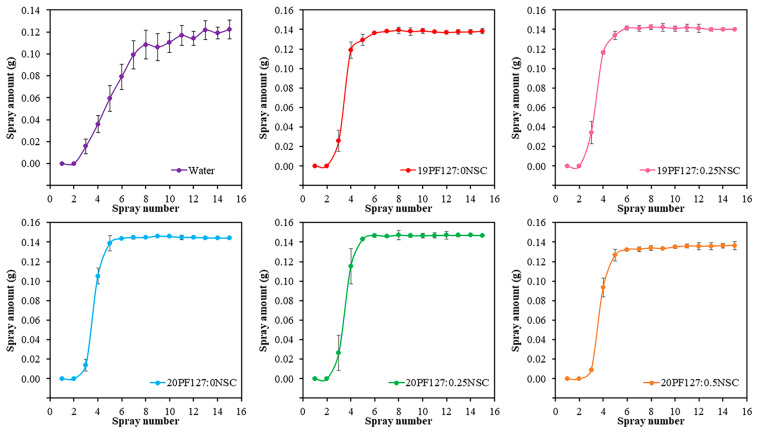
Sprayability of hydrogel solution with different concentrations of PF127 and NSC (*n* = 3, mean ± SD, *x*PF127:*y*NSC indicates a mixture with *x*% *w*/*v* PF127 and *y*% *w*/*v* NSC).

Sprayability was evaluated based on the amount of hydrogel solution released using a normal spray bottle. As shown in Figure 7, all hydrogel formulations exhibited consistent release volumes, with approximately 0.14 g of solution discharged from the nozzle after the fifth or sixth spray. This behavior aligns with previously reported findings [5]. Interestingly, compared to pure water, hydrogel formulations released slightly more mass per spray. This may be attributed to their higher viscosity, which influences the dynamics of spray ejection. Despite the increased viscosity, the hydrogel solutions remained sufficiently fluid to be atomized consistently and efficiently. All formulations containing PF127, with or without NSC, demonstrate a rapid increase in spray amount, typically reaching a plateau after five to six sprays. The cumulative amount stabilizes at around 0.14–0.16 g. Error bars are smaller than those in the water sample, indicating improved reproducibility and consistency.

**Figure 8:**
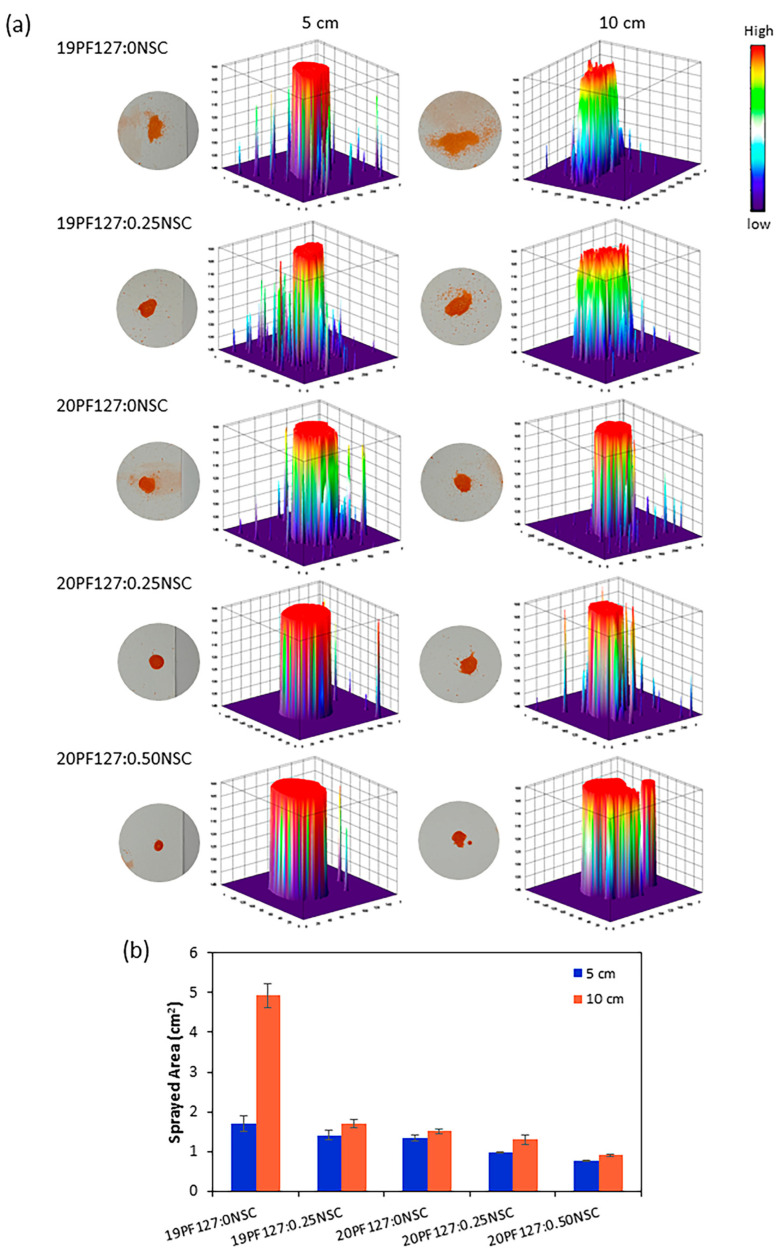
Spray characterization: (**a**) the effect of the spray distances on the spray thickness distribution, and (**b**) quantitative analysis of the sprayed area (*n* = 3, mean ± SD, *x*PF127:*y*NSC indicates a mixture with *x*% *w*/*v* PF127 and *y*% *w*/*v* NSC).

To further assess spray performance, spraying was conducted at two distances: 5 cm and 10 cm. The spray distributions of the PF127:NSC solution, which use single spraying delivered under various spray distances, were visualized with 3D graphs by ImageJ software version 1.54 (Figure 8a). It was found that spray distances affect spray thickness distributions. At 5 cm, the hydrogel was deposited more uniformly with a thicker and denser appearance, as indicated by darker color intensities in the 3D plots. In contrast, at 10 cm, the hydrogel spread over a broader area but formed a thinner layer, suggesting that increasing the spray distance promotes wider coverage but lower deposition density. Although only the spray distance was studied here, the viscosity of the spray solution is crucial for the distribution quality and droplet size formation. Higher viscosity formulations (with increased PF127 and/or NSC content) resulted in larger droplet sizes and a smaller surface area coverage. Quantitative analysis of the spray distribution (Figure 8b) revealed that the sprayed area ranged from 0.76 to 4.92 cm^2^, depending on spray distance and viscosity. Nevertheless, 19PF127:0NSC exhibited the largest spray area at a 10 cm spray distance, but the distribution was inconsistent, making the spray difficult to control. Therefore, optimal spray conditions were identified as involving shorter spray distances and lower viscosity, which promoted more uniform distribution across the targeted area.

#### 3.3.4. Gelling Capacity and Spreadability

The gelling capacity studies established that one drop of sol underwent gelation as soon as it was placed into solution at pH 7.4 at 34 ± 1 °C. The sol-to-gel transition occurred almost immediately and remained in gel form for >10 min, as shown in Table 2. This result confirms that the sprayable formulations rapidly gel at near skin temperature and maintain structural integrity for a sufficient period, indicating strong in situ gelling capacity.

The spreadability of the hydrogels ranged from 4.08 to 5.75 cm (Table 2), suggesting favorable spreading behavior. The gels were easily distributed across the glass surface with a smooth texture and no visible granularity. Moreover, the results revealed an inverse relationship between gelation strength and spreadability, consistent with prior research [30].

#### 3.3.5. Occlusive Property

The occlusive property can be very critical in terms of increasing skin hydration. Nonetheless, fully occlusive films can lead to skin irritation and promote microbial growth. Thus, formulations with non-occlusive to partially occlusive behavior are often preferred. The occlusivity factor (F) was used to evaluate the occlusive potential of the PF127:NSC sprayable hydrogels. It was found that F values for the five selected formulations ranged from 11.64 to 12.57. Specifically, values were 12.17 (19PF127:0NSC), 12.35 (19PF127:0.25NSC), 12.48 (20PF127:0NSC), 12.57 (20PF127:0.25NSC), and 11.64 (20PF127:0.50NSC). These values are comparable to those reported for non-occlusive or partially occlusive films (F ≈ 10.35) [31], indicating that the developed formulations offer appropriate occlusive behavior for safe topical application.

#### 3.3.6. Fluid Affinity

**Figure 9:**
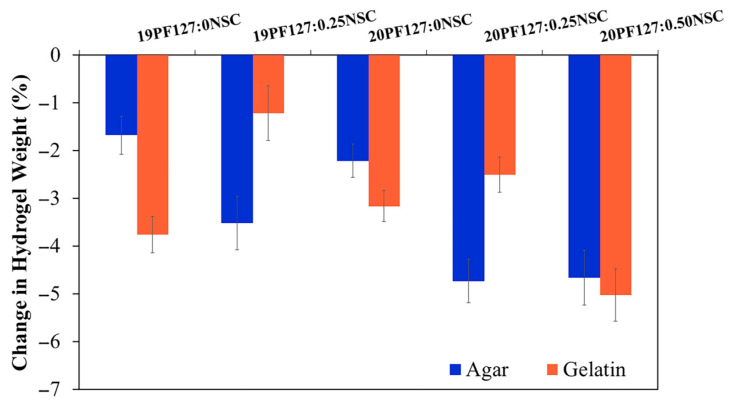
Fluid absorption and donation properties of the sprayable formulation (*n* = 3, mean ± SD, *x*PF127:*y*NSC indicates a mixture with *x*% *w*/*v* PF127 and *y*% *w*/*v* NSC).

A moist wound environment is widely regarded as optimal for wound healing. To encourage wound debridement and create a matrix for skin regeneration, it is essential to maintain a favorable moisture balance—to avoid an excessively wet or dry environment. The ability to donate fluid to wounds benefits autolytic debridement, while the ability to absorb wound exudate and debride slough benefits the re-epithelialization of healthy tissue. Overhydrating wounds can cause peri-wound tissue to macerate, which can prolong the healing process. Therefore, hydrogel dressings have to be able to absorb more moisture in excess exudative situations and provide moisture to dry wounds. To evaluate fluid affinity, water absorption and donation properties were assessed using agar and gelatin, following the standard “EN 13726-1:2002 test methods for primary wound dressings—Part 1: aspects of absorbency”. The ability of PF127:NSC spray formulations shows that a decreasing change in weight resulted in all spray formulations exhibiting a higher donation than absorption of liquids (Figure 9). All formulations exhibit negative changes in weight, indicating weight loss, which is generally attributed to water evaporation or fluid loss over time. Formulations without NSC (19PF127:0NSC and 20PF127:0NSC) show moderate weight loss at both temperatures. However, as PF127 concentration increases from 19% to 20%, slightly greater weight loss is observed, possibly due to differences in network density or initial water content. The addition of NSC further increases weight loss. Notably, 20PF127:0.50NSC exhibits the highest weight loss among all formulations, with over 6% reduction. Higher NSC content may increase hydrophilicity and fluid affinity, leading to higher initial water uptake but also greater susceptibility to evaporation under thermal conditions. Considering that the target wound type for the spray format was dry to low-exuding wounds, the resulting PF127:NSC spray formulations in this study were considered a positive outcome, showing good buffering capacity for moisture handling in the wound bed.

### 3.4. Short Term Stability of Solution Studies

**Figure 10:**
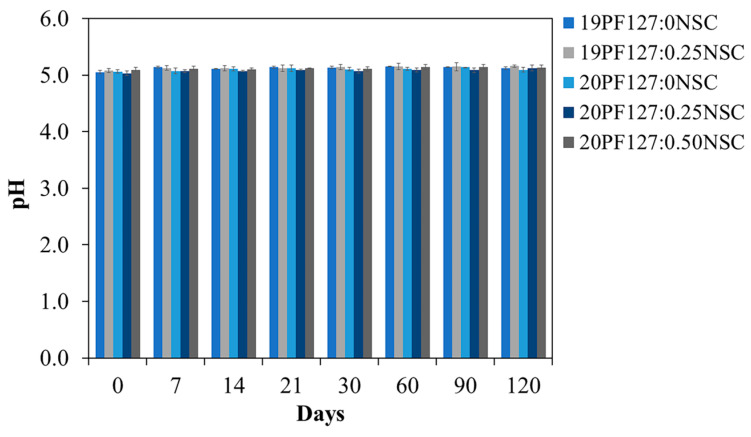
pH stability of the hydrogel spray solution after 120 days of storage at 4 °C (*n* = 3, mean ± SD, *x*PF127:*y*NSC indicates a mixture with *x*% *w*/*v* PF127 and *y*% *w*/*v* NSC).

Data obtained for short-term stability studies of the formulation, which was stored at 4 °C for 120 days, is presented in terms of clarity, gelation time, gelling capacity and spreadability (Table 2). The formulation kept at 4 °C for 60 and 120 days after the initially data recording did not exhibit any change concerning the clarity and color of the preparation. There also was no sign of precipitation in the stored spray solutions, and no change was seen in the viscosity of the formulation. A slight increase in gelation time was recorded over the period of storage for some formulations (19PF127:0NSC, 20PF127:0NSC, and 20PF127:0.25NSC), but this change remained within acceptable limits for clinical application. However, the 20PF127:0.50NSC formulation exhibited a noticeable increase in gelation time, while 19PF127:0.25NSC completely lost its ability to form a gel, suggesting a decline in thermo-responsive performance. Considering the effect of storage stability on the gelling capacity results, 19PF127:0NSC and 20PF127:0.50NSC solutions exhibited changes after 60 days of storage. Both formulations initially formed gels when dropped into a pH 7.4 solution at 35 ± 1 °C but dissolved rapidly within a few minutes. Minor variations were also observed in spreadability results for certain formulations after long-term storage. In addition, pH measurements showed a slight but statistically insignificant increase (*p* > 0.05) during the first 7 days, with no further changes detected over the remaining study period (Figure 10). Over the period of the experiment, the formulations 20PF127:0NSC and 20PF127:0.25NSC demonstrated good chemical and physical stability for at least 120 days when stored at 4 °C, supporting their suitability for further development as sprayable wound dressings.

Key criteria for selecting the optimal sprayable hydrogel formulation included good sprayability, a gelation time of approximately 1 min, a gelation temperature below 35 °C, effective liquid and moisture management, and long-term stability when stored at 4 °C. 20PF127:0NSC and 20PF127:0.25NSC possess these properties. Both exhibit a non-flowing gel state at 35 ± 1 °C, which corresponds to the typical surface temperature of human skin. This ensures that the formulation can be applied as a spray that rapidly transitions into a stable gel upon contact with the wound site. Hence, 20PF127:0NSC and 20PF127:0.25NSC were suggested as the representative hydrogel formulations for further investigation as drug delivery vehicles.

### 3.5. Cytotoxicity Assay

**Figure 11:**
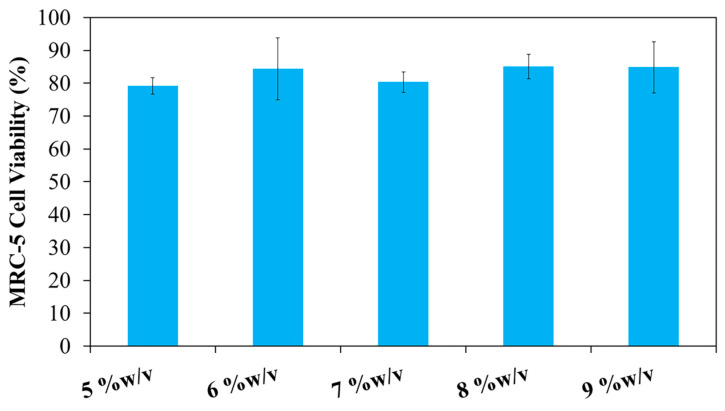
In vitro cytotoxicity of 20PF127:0.25NSC at different concentrations on the viability of MRC-5 cell lines as measured by MTT assay.

This study aimed to develop a sprayable hydrogel incorporating both thermo- and pH-responsive polymers. The formulation designated as 20PF127:0.25NSC was selected for further investigation due to its favorable physicochemical properties during prolonged storage compared to other formulations. This hydrogel consists of PF127 as a thermo-responsive polymer and NSC as a pH-responsive polymer. Therefore, this formulation was used to evaluate the effect of hydrogel composition on biocompatibility. The cytotoxicity of the PF127:NSC sprayable hydrogel was evaluated using an MTT assay on MRC-5 human fibroblast cells. The sprayable hydrogel solution at a concentration of 20% *w*/*v* could not completely dissolve in the medium at 37 °C, necessitating the use of a serial dilution. As a result, lower concentrations of 5, 6, 7, 8, 9, and 10% *w*/*v* were prepared for cytotoxicity evaluation (Figure 11). Cell viability remained above 80% for PF127:NSC hydrogel concentrations of 5, 6, 7, 8, and 9% *w*/*v*, indicating good biocompatibility. According to ISO 10993-5 standards, materials that maintain cell viability above 70% are considered non-cytotoxic [32,33]. Consequently, based on the percentage of viable cells, the PF127:NSC hydrogel can be considered non-cytotoxic at concentrations ≤9% *w*/*v*. These results underscore the potential of this formulation for safe use as a wound dressing in biomedical applications.

### 3.6. In Vitro Drug Release Studies

The 20PF127:0.25NSC hydrogel was chosen for investigation as a drug delivery vehicle, with neem extract incorporated as the bioactive agent. *Azadirachta indica* (neem) has been extensively utilized in traditional medicine systems for its therapeutic potential in wound healing. Its efficacy is attributed to a range of bioactive constituents, including nimbidin, nimbolide, and azadirachtin, which exhibit potent antibacterial, antifungal, anti-inflammatory, antioxidant, and immunomodulatory properties. These phytochemicals contribute to reduced microbial load, attenuation of the inflammatory response, and mitigation of oxidative stress—all of which are critical in the wound healing process. Furthermore, neem has been shown to enhance collagen synthesis, accelerate wound contraction, and facilitate re-epithelialization, thereby promoting faster tissue repair and regeneration. A growing body of preclinical and clinical evidence supports its application as a complementary therapeutic agent in wound management [34,35,36,37,38]. Therefore, neem was incorporated into a sprayable hydrogel in this research to enhance its effectiveness for wound healing by studying its release behavior and antioxidant properties.

The physical entrapment of drug molecules in micellar aggregates is due to the existence of intermolecular interactions: hydrophobic, van der Waals interactions, or hydrogen bonds. In the case of neem extract, the chemical structure of its bioactive compounds suggests that hydrogen bonding—particularly between the ether oxygen atoms of the PEO chains in PF127 and the carbonyl and hydroxyl groups of the bioactive molecules—plays a key role in drug encapsulation. These hydrogen bonds act in addition to the van der Waals interactions occurring within the hydrophobic PPO core of the micelles, enhancing the overall encapsulation efficiency [39]. The amount of neem incorporated into the PF127:NSC hydrogel, expressed as drug loading (DL) and entrapment efficiency (EE), is presented in Figure S1. As the initial concentration of neem extract added to the hydrogel increased from 0.05 to 0.30 g/mL, the DL values also increased significantly, ranging from 21.79 to 59.18%. The EE reached 89% at an initial neem concentration of 0.05 g/mL and increased slightly to approximately 96% at 0.10 g/mL. Beyond this concentration (0.20–0.30 g/mL), EE values plateaued with no significant difference observed (*p* > 0.05), indicating saturation of the encapsulation capacity of the PF127:NSC hydrogel system. Furthermore, the addition of the bioactive agent affected both the gelation temperature (T_gel_) and gelation time, consistent with previous reports [40]. In this study, the incorporation of neem extract—particularly at higher concentrations—resulted in a decrease in both T_gel_ and gelation time (Figure S2). This behavior is attributed to the increased hydrophobicity of the system, as hydrophobic groups from the plant extract promote micelle aggregation and quickened gel formation with rising temperatures [41].

The release mechanism of neem from neem-loaded 20PF127:0.25NSC hydrogels was investigated at 37 °C in phosphate-buffered saline (PBS, pH 7.4). Although in vitro drug release from topical formulations is often performed at 35 ± 1 °C to mimic the natural skin conditions, these studies were carried out at 37 °C to ensure that the formulation transitions from sol to complete gel form. PBS solution at pH 7.4 was used as the release medium to simulate the physiological environment of wounded skin, which generally has a pH between 6.5 and 8.9. Wounded skin typically has an alkaline pH, with deeper wounds showing higher pH levels than shallow wounds. As healing progresses, the pH gradually decreases, returning to the acidic range of healthy skin (pH 4.0–6.0) [29]. 

**Figure 12:**
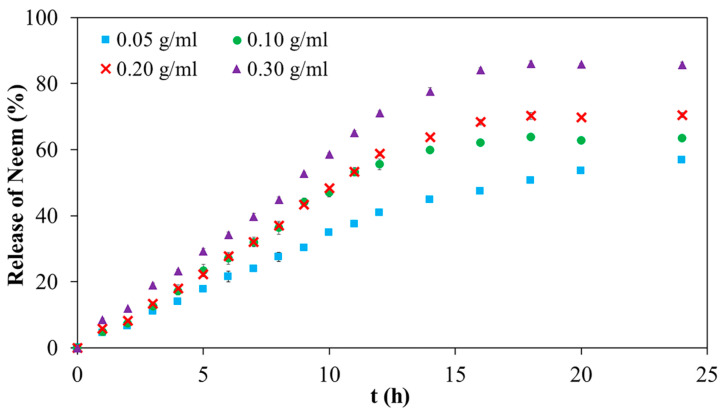
Neem release profile of neem-loaded 20PF127:0.25NSC sprayable hydrogel.

The release profile of neem extract from 20PF127:0.25NSC hydrogels containing various concentrations of neem (0.05, 0.10, 0.20, and 0.30 g/mL) is shown in Figure 12. All formulations displayed a biphasic release pattern characterized by an initial burst release within the first 12 h, followed by a slower, sustained release phase thereafter. The overall cumulative percentage drug diffusion for 20PF127:0.25NSC hydrogel, incorporating different neem concentrations with 0.05, 0.10, 0.20, and 0.30 g/mL, was found to be 57, 64, 70, and 86%, respectively. The increase in neem concentration led to a corresponding increase in diffusion rate. For the sprayable solution of the neem-loaded hydrogel, the neem compound was entrapped inside the hydrophobic core of the micelles, and some free neem compound diffused in the free volume of the hydrogel network, affecting release behavior. The observed lower drug release suggests that entrapment within the micelles contributed significantly to the limited release of the compound, whereas the higher concentrations resulted in more free, unentrapped neem molecules in the hydrogel network, resulting in more release. In addition, the neem compound affected the reduction of the packed micellar aggregates of PF127, which could indicate that the neem inclusion led to a weaker gel network at high neem content. This weaker network would make it easier for micelles to diffuse in the medium. As a result, the hydrogel containing 0.30 g/mL of neem showed a larger cumulative percentage release than the hydrogel containing 0.05 g/mL of neem. These results demonstrate the tunability of neem release profiles by adjusting initial neem concentrations in the PF127:NSC hydrogel matrix. Such control allows customization of wound dressings to different wound types and healing stages, improving their therapeutic effectiveness.

In principle, there are two ways for drugs to be released from micelle-hydrogel systems. One is that the drug molecules are released from the micelles in the hydrogel network compartment and then diffuse into the dissolution medium; the other is that drug-loaded micelles either directly diffuse into the medium through an aqueous channel and then release the encapsulated drug molecules. To better understand the mechanisms governing the release of the bioactive agent, the release profiles were analyzed using empirical mathematical models, including the Korsmeyer–Peppas, zero-order, and Hixson–Crowell models, as described in the Supplementary Materials. The Korsmeyer–Peppas model was employed to describe drug release from a polymeric system when the exact release mechanism is unknown or involves multiple concurrent processes. This model was also used to distinguish between diffusion-controlled, relaxation-controlled, and degradation-controlled release mechanisms. Table S1 presents the parameters obtained from model fitting. Among the models tested, the Korsmeyer–Peppas model provided the best fit to the experimental data, with correlation coefficients (*r*^2^) exceeding 0.988. The release exponent *n* values ranged from 0.89 to 1.01 and increased with the concentration of neem. These values suggest a non-Fickian diffusion mechanism (Super Case II) involving a combination of polymer chain relaxation and matrix erosion. This indicates that, in addition to diffusion, other factors related to the structural characteristics and nature of the hydrogels influence the release process. The release of ciprofloxacin from Pluronic F127 and F68 hydrogels has been reported to follow zero-order kinetics, indicating a constant release rate independent of drug concentration [3]. Similarly, lidocaine released from F127-based hydrogels also exhibited zero-order behavior; however, further analysis using the Korsmeyer–Peppas model revealed a super case-II transport mechanism, suggesting that the release was governed by a combination of diffusion and erosion-controlled processes [42]. To further investigate the release behavior, the zero-order and Hixson–Crowell models were also applied, as these models consider constant release rates and changes in surface area and matrix structure, respectively—factors relevant to non-Fickian diffusion and erosion-based mechanisms. All materials exhibited a relatively strong fit to the zero-order model, with correlation coefficients around 0.996, while the Hixson–Crowell model showed a slightly lower fit, with *r^2^* values near 0.970 (Table S1). Both models reinforce our previous analysis of the effect of the erosion on the matrix on the release process. Taken together, the modelling results suggest that in the neem-loaded hydrogels studied, drug release is primarily governed by the rate of micellar dissociation, which weakens the gel network and facilitates the diffusion of the bioactive agent [43,44,45].

### 3.7. Antioxidant Activity

**Figure 13:**
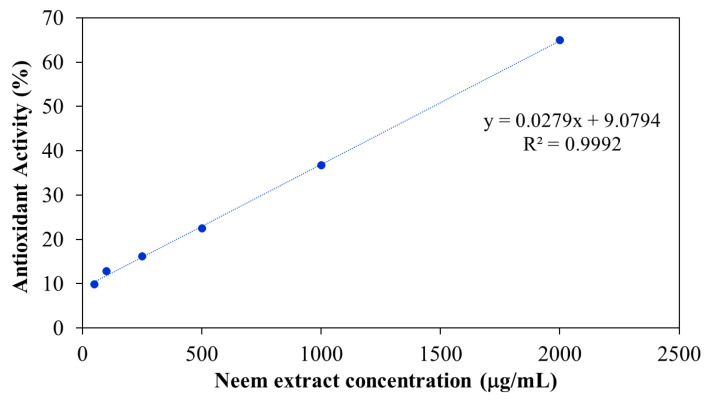
Antioxidant activity of aqueous neem extract.

**Figure 14:**
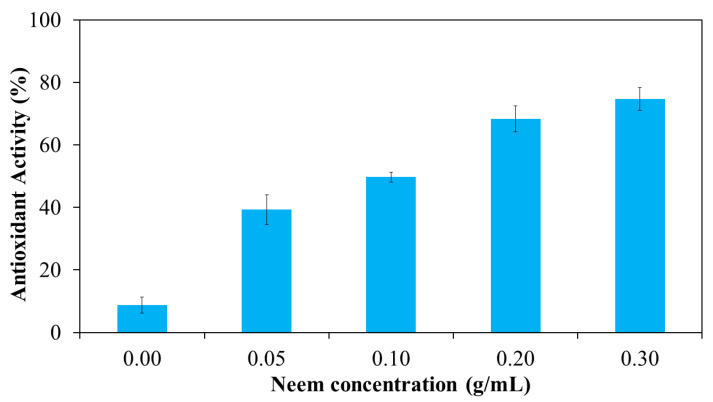
Antioxidant activity of neem-loaded PF127:NSC sprayable hydrogel.

Free radicals play a key role in the pathogenesis of numerous diseases, making their neutralization a critical strategy for disease prevention. Antioxidants function by donating electrons to free radicals, thereby stabilizing or deactivating them before they can damage cellular components. One widely accepted method for evaluating antioxidant activity is the DPPH assay, which is based on the ability of antioxidants to reduce the stable DPPH radical to a non-radical form. The results in Figure 13 shown the antioxidant activity of aqueous neem extract with various concentrations. The result revealed that percentage inhibition ranged from 10% at 50 μg/mL to 65% at 2000 μg/mL. The highest antioxidant activity was observed at 2000 μg/mL, with decreasing activity at lower concentrations. The antioxidant activity of the neem-loaded PF127:NSC hydrogels was also evaluated using the DPPH method (Figure 14). The control hydrogel without neem extract exhibited low antioxidant activity (~9%). Conversely, neem-loaded hydrogels displayed significantly enhanced antioxidant activity, with inhibition rates of 39, 50, 68, and 75% corresponding to neem concentrations of 0.05, 0.10, 0.20, and 0.30 g/mL, respectively. The results show a clear dose-dependent enhancement in radical scavenging activity with increasing neem concentration. This improvement is likely attributed to the presence of polyphenolic and flavonoid compounds in neem, which are known for their effectiveness in scavenging reactive oxygen species [46,47,48]. These findings suggest that PF127:NSC hydrogels incorporated with neem extract could effectively reduce oxidative stress in wound environments, thereby promoting faster healing and reducing inflammation.

## 4. Conclusions

Sprayable hydrogels offer significant promise as transdermal systems for skin wound treatment due to their ease of application and adaptability to irregular wound surfaces. In this study, a novel sprayable hydrogel formulation incorporating neem extract was successfully developed using Pluronic F127 (PF127) and *N*-succinyl chitosan (NSC) as thermo- and pH-responsive polymers, respectively. NSC was synthesized through the chemical modification of chitosan with succinic anhydride. A succinic anhydride-to- chitosan molar ratio of 5:1 yielded the highest degree of substitution, producing a water-soluble polymer effective across a broad pH range. The investigation of five sprayable hydrogel formulations (19PF127:0NSC, 19PF127:0.25NSC, 20PF127:0NSC, 20PF127:0.25NSC, and 20PF127:0.50NSC) demonstrated that the concentrations of both PF127 and NSC significantly influenced the sol–gel transition behavior, sprayability and gelation properties. Among these, the optimal formulation, comprising 20% *w*/*v* PF127 and 0.25% *w*/*v* NSC (20PF127:0.25NSC), exhibited favorable sol–gel transition behavior, excellent sprayability, and desirable gel properties. The precursor solution exhibited appropriate flow characteristics, enabling consistent and convenient spray administration. The optimized hydrogel formulation also demonstrated satisfactory physicochemical properties, including excellent clarity, pH compatibility, rapid gelation (~1 min), in situ gelation at physiological temperature (35 °C), as well as effective liquid and moisture management. Furthermore, the non-occlusive to partially occlusive properties of the sprayable hydrogel contributed to efficient moisture control, supporting a favorable wound healing environment. These formulations also hold potential as user-friendly dressings for dry to low-exuding wounds, such as those commonly arising from sports injuries and inflammatory joint conditions. In vitro MTT assay results confirmed that the PF127:NSC hydrogel was non-cytotoxic to MRC-5 fibroblast cells, supporting its biocompatibility for topical applications. Moreover, the hydrogel remained stable for at least four months under refrigerated storage conditions (4 °C), with no observable changes in pH, precipitation, or gelation performance. Various concentrations of neem extract (0.05–0.30 g/mL) were incorporated into the sprayable hydrogel formulation to identify the concentration with most suitable gelation properties and antioxidant activity. Both the percentage of neem release and antioxidant activity increased with higher neem content. The incorporation of neem extract into the sprayable hydrogel yielded a sustained drug release profile, with 56–86% of neem released over 24 h, depending on the concentration of loaded neem. Furthermore, the neem-loaded hydrogel spray demonstrated significantly higher antioxidant activity in the DPPH assay (39–75%) compared to the hydrogel without neem (9%). Collectively, this study successfully established a multifunctional sprayable hydrogel system based on PF127 and NSC that is both pH- and thermo-responsive. These hydrogels demonstrate promising potential for wound dressing applications and as an effective delivery system for neem extract.

## Figures and Tables

**Table 1:** The degree of substitution and solubility at various pH levels of chitosan and NSC products.

Samples	Molar Ratios (SA:CS *)	DS (%)	Solubility					
			pH 4	pH 5	pH 6	pH 7	pH 8	pH 9
CS	0	-	+++	--	--	--	--	--
NSC-1	1:1	11.3	+++	--	--	--	--	--
NSC-3	3:1	37.3	+++	+++	--	--	++	--
NSC-5	5:1	57.2	+++	+++	--	--	+++	+++

* Moles of glucosamine unit (or per NH_2_ group). +++ soluble, ++ partially soluble, and -- insoluble.

**Table 2:** Physicochemical characterization and stability results of sprayable hydrogel formulation (*n* = 3, mean ± SD, *x*PF127:*y*NSC indicates a mixture with *x*% *w*/*v* PF127 and *y*% *w*/*v* NSC).

Days	Samples	Clarity	Gelation Time * (s ± SD)	Gelling Capacity	Spreadability (cm ± SD)
0	19PF127:0NSC	+++	81.5 ± 8.6	++	4.36 ± 0.08
	19PF127:0.25NSC	+++	362.5 ± 3.5	++	5.68 ± 0.14
	20PF127:0NSC	+++	44.4 ± 5.5	++	4.12 ± 0.07
	20PF127:0.25NSC	+++	55.3 ± 1.5	++	4.42 ± 0.02
	20PF127:0.50NSC	++	79.5 ± 1.8	++	4.64 ± 0.06
60	19PF127:0NSC	+++	81.6 ± 2.3	+	4.33 ± 0.06
	19PF127:0.25NSC	+++	NG	-	-
	20PF127:0NSC	+++	46.9 ± 3.2	++	4.23 ± 0.06
	20PF127:0.25NSC	+++	61.5 ± 1.6	++	5.10 ± 0.10
	20PF127:0.50NSC	++	125.3 ± 5.3	+	5.68 ± 0.03
120	19PF127:0NSC	+++	83.6 ± 2.9	+	4.43 ± 0.05
	19PF127:0.25NSC	+++	NG	-	-
	20PF127:0NSC	+++	48.6 ± 3.7	++	4.27 ± 0.14
	20PF127:0.25NSC	+++	64.5 ± 4.2	++	5.12 ± 0.16
	20PF127:0.50NSC	++	123.9 ± 9.9	+	5.75 ± 0.02

* = 37 °C. NG = no gelation.

## Data Availability

The original contributions presented in this study are included in the article/Supplementary Materials. Further inquiries can be directed to the corresponding authors.

## Supplementary Materials

The following supporting information can be downloaded at: https://www.mdpi.com/article/10.3390/polym17152157/s1, Figure S1: Drug loading (DL) and entrapment efficiency (EE) for PF127:NSC sprayable hydrogel with neem loading; Figure S2: Gelation temperature and gelation time (at 37 °C) for PF127:NSC sprayable hydrogel with neem loading; Figure S3: The Korsmeyer-Peppas, zero-order, and Hixson-Crowell models for PF127:NSC sprayable hydrogel with neem loading; Table S1: Release kinetic of neem release from PF127:NSC sprayable hydrogels.
