# Supplementary material for: Design and Development of a Sprayable Hydrogel Based on Thermo/pH Dual-Responsive Polymer Incorporating Azadirachta indica (Neem) Extract for Wound Dressing Applications
Source: Polymers (Basel). 2025 Aug 7;17(15):2157. doi: 10.3390/polym17152157 (PMC12349343; doi:10.3390/polym17152157)
Supplement: Supplementary file 1 [file polymers-17-02157-s001.zip › polymers-3793473-supplementary.pdf]

Supplementary Materials

Design and Development of a Sprayable Hydrogel Based on Thermo/pH Dual-Responsive Polymer Incorporating of *Azadirachta indica* (Neem) Extract for Wound Dressing Applications

Amlika Rungrod [1], Arthit Makarasen [1], Suwicha Patnin [1], Supanna Techasakul [1,*] and Runglawan Somsunan [2,3,*]

[1] Laboratory of Organic Synthesis, Chulabhorn Research Institute, Bangkok, 10210, Thailand
amlika@cri.or.th (A.R.); arthit@cri.or.th (A.M.); suwicha@cri.or.th (S.P.)
[2] Department of Chemistry, Faculty of Science, Chiang Mai University, Chiang Mai, 50200, Thailand
[3] Center of Excellence in Materials Science and Technology, Chiang Mai University, Chiang Mai, 50200, Thailand
* Correspondence: runglawan.s@cmu.ac.th (R.S.) and supanna@cri.or.th (S.T.)

The drug loading (DL) and entrapment efficiency (EE) were calculated using the following equations:

DL (%) = (Weight of the drug in hydrogel solution / weight of polymer + drug) × 100

EE (%) = (Weight of the drug in hydrogel solution / weight of theoretical drug) × 100

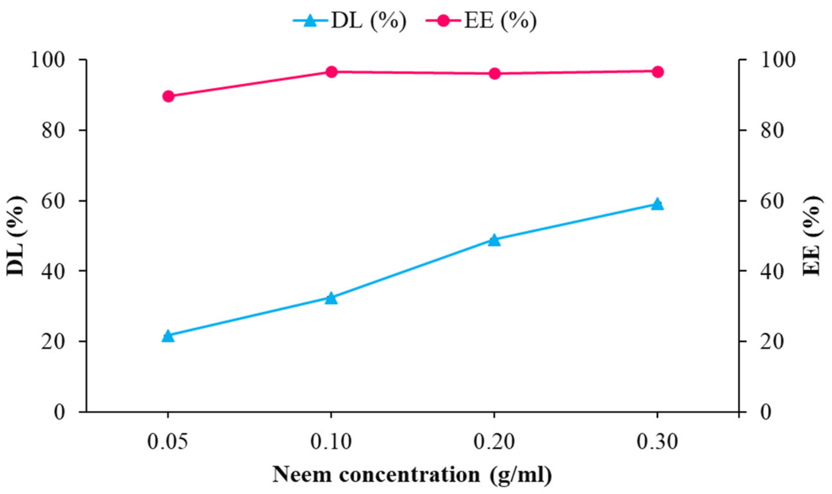

Figure S1 Drug loading (DL) and entrapment efficiency (EE) for PF127:NSC sprayable hydrogel with neem loading.

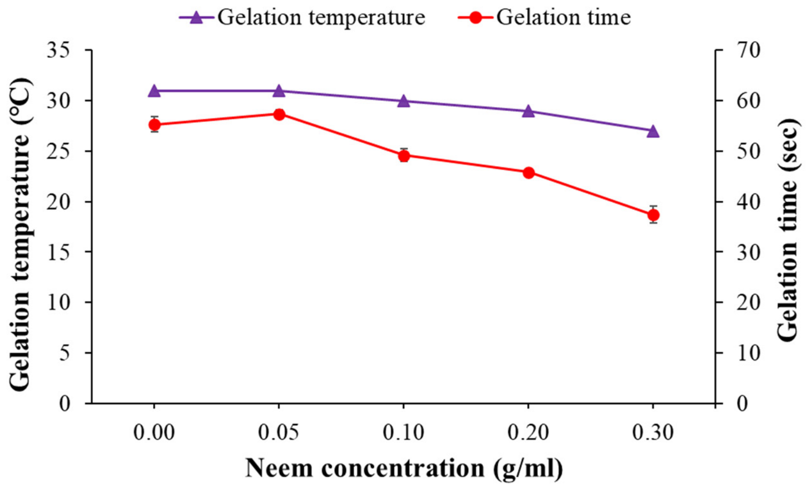

Figure S2 Gelation temperature and gelation time (at 37 °C) for PF127:NSC sprayable hydrogel with neem loading.

To better understand the mechanisms governing the release of the bioactive agent, the release profiles were analyzed using empirical mathematical models, including the Korsmeyer-Peppas, zero-order, and Hixson-Crowell models

The following equation was used to represent the Korsmeyer-Peppas model:

$$log\frac{Q_t}{Q_o} = log\ K_{KP} + n\ log\ t$$

Zero-order model:

$$\frac{Q_t}{Q_o} = K_o\ t$$

Hixson-Crowell model:

$$Q_o^{1/3} - Q_1^{1/3} = K_{HC}\ t$$

where Q_t represents the amount of drug released at the time (t) and Q_0 the amount of drug loaded, K is the constant of incorporation of structural modifications and geometrical characteristics of the system (also considered as the release velocity constant), and n is the release exponent (related to the drug release mechanism). The n values lower than 0.5 indicate a drug release profile controlled by classical diffusion; for values between 0.5 and 1, the drug release is of the anomalous type (a combination of classical diffusion with the relaxation mechanism of the matrix). Finally, when the values are reaches 1 and above, the release of the drug is subject to the relaxation of the polymeric chains and erosion of matrix.

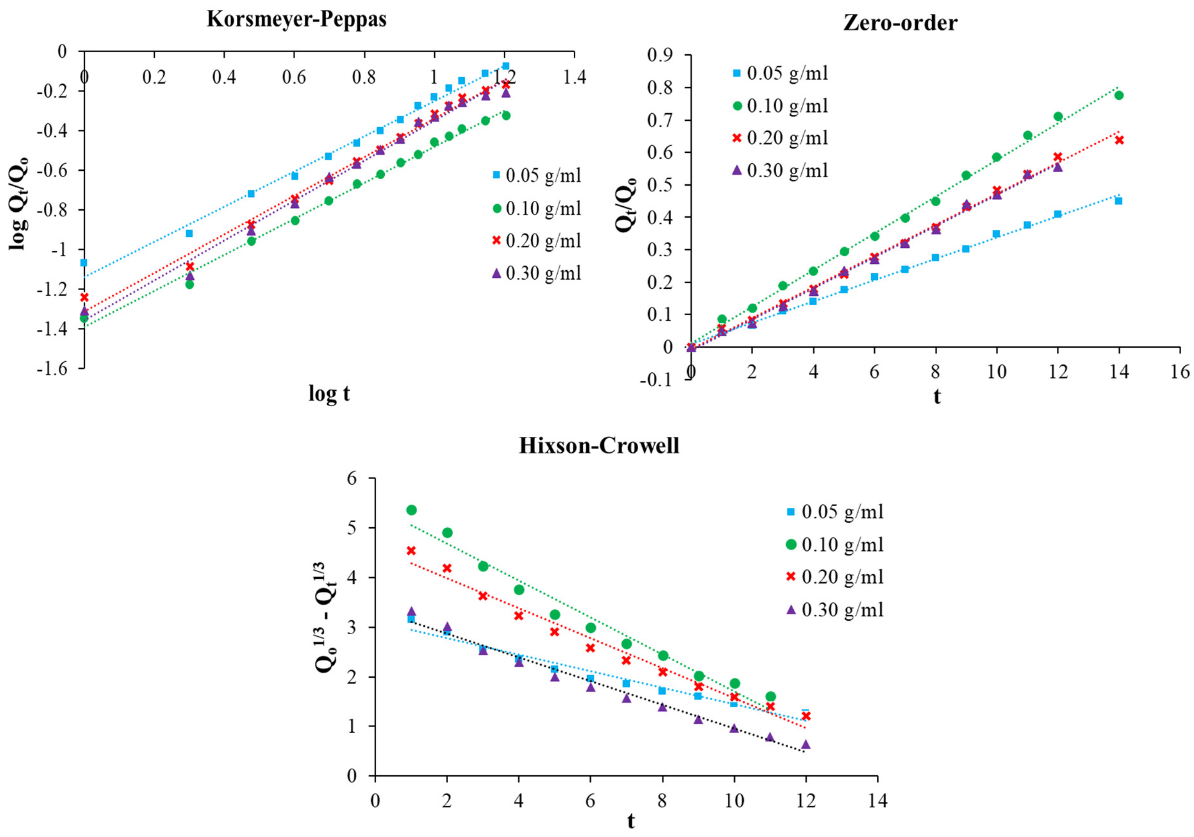

Figure S3 The Korsmeyer-Peppas, zero-order, and Hixson-Crowell models for PF127:NSC sprayable hydrogel with neem loading.

Table S1 Release kinetic of neem release from PF127:NSC sprayable hydrogels.

Neem concentration (g/ml)	Korsmeyer-Peppas			Zero-order		Hixson-Crowell	
	r^2	K_{KP}	n	r^2	K_0	r^2	K_{HC}
0.05	0.991	0.073	0.889	0.997	0.033	0.962	-0.166
0.10	0.994	0.041	0.911	0.997	0.057	0.979	-0.239
0.20	0.990	0.049	0.975	0.996	0.048	0.977	-0.301
0.30	0.989	0.044	1.008	0.996	0.048	0.971	-0.372
